# α-Thalassemia Impairs the Cytoadherence of *Plasmodium falciparum*-Infected Erythrocytes

**DOI:** 10.1371/journal.pone.0037214

**Published:** 2012-05-18

**Authors:** Michael A. Krause, Seidina A. S. Diakite, Tatiana M. Lopera-Mesa, Chanaki Amaratunga, Takayuki Arie, Karim Traore, Saibou Doumbia, Drissa Konate, Jeffrey R. Keefer, Mahamadou Diakite, Rick M. Fairhurst

**Affiliations:** 1 Laboratory of Malaria and Vector Research, National Institute of Allergy and Infectious Diseases, National Institutes of Health, Bethesda, Maryland, United States of America; 2 Malaria Research and Training Center, Faculty of Medicine, Pharmacy, and Odontostomatology, University of Bamako, Bamako, Mali; 3 Department of Physics and Electronics, School of Engineering, Osaka Prefecture University, Osaka, Japan; 4 Division of Pediatric Hematology, Department of Pediatrics, Johns Hopkins School of Medicine, Baltimore, Maryland, United States of America; Bernhard Nocht Institute for Tropical Medicine, Germany

## Abstract

**Background:**

α-thalassemia results from decreased production of α-globin chains that make up part of hemoglobin tetramers (Hb; α_2_β_2_) and affects up to 50% of individuals in some regions of sub-Saharan Africa. Heterozygous (−α/αα) and homozygous (−α/−α) genotypes are associated with reduced risk of severe *Plasmodium falciparum* malaria, but the mechanism of this protection remains obscure. We hypothesized that α-thalassemia impairs the adherence of parasitized red blood cells (RBCs) to microvascular endothelial cells (MVECs) and monocytes – two interactions that are centrally involved in the pathogenesis of severe disease.

**Methods and Findings:**

We obtained *P. falciparum* isolates directly from Malian children with malaria and used them to infect αα/αα (normal), −α/αα and −α/−α RBCs. We also used laboratory-adapted *P. falciparum* clones to infect −/−α RBCs obtained from patients with HbH disease. Following a single cycle of parasite invasion and maturation to the trophozoite stage, we tested the ability of parasitized RBCs to bind MVECs and monocytes. Compared to parasitized αα/αα RBCs, we found that parasitized −α/αα, −α/−α and −/−α RBCs showed, respectively, 22%, 43% and 63% reductions in binding to MVECs and 13%, 33% and 63% reductions in binding to monocytes. α-thalassemia was associated with abnormal display of *P. falciparum* erythrocyte membrane protein 1 (PfEMP1), the parasite’s main cytoadherence ligand and virulence factor, on the surface of parasitized RBCs.

**Conclusions:**

Parasitized α-thalassemic RBCs show PfEMP1 display abnormalities that are reminiscent of those on the surface of parasitized sickle HbS and HbC RBCs. Our data suggest a model of malaria protection in which α-thalassemia ameliorates the pro-inflammatory effects of cytoadherence. Our findings also raise the possibility that other unstable hemoglobins such as HbE and unpaired α-globin chains (in the case of β-thalassemia) protect against life-threatening malaria by a similar mechanism.

## Introduction

α-thalassemia is an inherited disorder of hemoglobin (Hb) synthesis, in which reduced production of α-globin chains leads to decreased amounts of normal α_2_β_2_ tetramers and increased amounts of unpaired β-globin chains. In sub-Saharan Africa, α-thalassemia states are produced by a 3.7-kb deletion that leaves one functional copy of duplicated α-globin genes. Heterozygotes (−α/αα) have an essentially normal phenotype while homozygotes (−α/−α) have mild microcytic anemia. HbH disease is a chronic hemolytic disorder that may produce severe anemia requiring periodic blood transfusions. While the prevalence of α-thalassemia can exceed 50% in some malaria-endemic areas of sub-Saharan Africa, HbH disease (−/−α) is extremely rare because the mutant *cis* allele (−/) is very uncommon.

One study associated α-thalassemia with reduced risk of severe *Plasmodium falciparum* malaria in Papua New Guinea [1], but only recently have epidemiological studies confirmed this finding in sub-Saharan Africa. Case-control and cohort studies have shown that heterozygous and homozygous α-thalassemia variously protect against severe malaria syndromes, including cerebral malaria (CM; coma with or without convulsions), severe malarial anemia (SMA; Hb level ≤50 g/l), and deep acidotic breathing [2], [3], [4], [5]. While these observations provide strong evidence that α-thalassemia prevents the progression from uncomplicated to severe malaria, the mechanism of this protection has not been established.

In considering candidate mechanisms of protection by α-thalassemia, we reasoned that they should be consistent with a well-established epidemiological observation reported from numerous African settings. Specifically, α-thalassemia is not associated with reduced parasite prevalence [5], [6], [7] or densities *in vivo* as determined by examining blood smears from children with asymptomatic parasitemia [2], [8], uncomplicated malaria [2], [3], [4], [5], [6], [7], [9], [10], [11], [12], or severe malaria [2], [13]. As noted by other investigators [3], [5], [14], these observations indicate that α-thalassemia does not protect against severe malaria by mechanisms that impair the ability of parasites to invade or develop within RBCs [15], [16], or promote the removal of parasitized RBCs from the bloodstream by increased neoantigen expression [15], [17] or increased antibody binding [18]. Indeed, we have observed extremely high parasite burdens (up to 200,000/µl) in some −α/αα and −α/−α children with malaria (unpublished data).

A central question thus emerges from these considerations: how can marginal decreases in α-globin synthesis enable children to develop high parasite densities and symptoms of uncomplicated malaria and yet not develop severe complications of the disease? To explore this question, we hypothesized that α-thalassemia impairs the adherence of parasitized RBCs to microvascular endothelial cells (MVECs) and monocytes. These cell-cell interactions are mediated by *P. falciparum* erythrocyte membrane protein 1 (PfEMP1), a family of clonally-expressed, antigenically-variant proteins [23], [24], [25]. *P. falciparum* parasites export PfEMP1 proteins and concentrate them in knob-like protrusions on the surface of their host RBCs. This host cell modification enables a large mass of parasitized RBCs to sequester in venous microvessels [26] and avoid clearance from the bloodstream by the spleen. However, the adherence of parasitized RBCs to MVECs and monocytes also contributes to the development of life-threatening malaria by causing systemic microvascular inflammation [27], [28], [29], [30].

Using naturally-circulating *P. falciparum* isolates and freshly-drawn RBCs from our study site in rural Mali, where approximately 40% of children are α-thalassemic, we show that α-thalassemia impairs the cytoadherence of parasitized RBCs and is associated with abnormal PfEMP1 display.

## Methods

### Red Blood Cells

All blood samples were obtained from healthy Malian children who were confirmed to be aparasitemic by examination of thick blood smears. Blood samples from two patients with HbH disease were obtained in the United States. Written, informed consent was obtained from all blood donors or their parents. Blood collection was approved by the Institutional Review Boards of the National Institute of Allergy and Infectious Diseases, the University of Bamako, and the Johns Hopkins School of Medicine. Whole blood samples were drawn into Vacutainers® (Becton-Dickinson, Franklin Lakes, NJ) containing acid-citrate-dextrose (ACD) anticoagulant. After removing buffy coat leukocytes, RBCs were washed three times with RPMI-1640 (Invitrogen, Carlsbad, CA) and stored at 50% hematocrit at 4°C prior to use (within 4–36 h of blood draw). In some flow cytometry experiments, whole blood samples were stored in ACD Vacutainers® on ice during a 36-hour transport from Mali to the United States and then used within 4–24 h of arrival. Hemoglobin types were determined by HPLC (D-10 Instrument, Bio-Rad, Hercules, CA). The presence of the G6PD*A− allele was determined as described [19]. The presence of the 3.7-kb deletional determinant of α-thalassemia was determined as described [20]. In all experiments, normal and α-thalassemic RBCs were obtained simultaneously and processed in parallel. Normal and α-thalassemic RBC donors from Mali had normal HbA and did not carry the G6PD*A− allele.

### Parasite Culture

*P. falciparum* clones (3D7, FCR-3, A4tres and FVO) were cultured in O+ RBCs at 5% hematocrit in complete medium (CM; RPMI-1640 supplemented with 25 mg/ml HEPES, 2 mg/mL sodium bicarbonate, 50 µg/ml gentamicin, and 0.5% Albumax II (Gibco-BRL, Grand Island, NY) in 0.2 µm-vented, 75-cm^2^ flasks (Corning Inc., Corning, NY). Knobby parasite lines were maintained by periodic gelatin flotation [21]. Cultures were maintained at 37°C in a humidified atmosphere of 5% CO_2_ and media was changed every 8–12 h. Naturally-circulating *P. falciparum* isolates (ring stage) were obtained directly from Malian children with malaria and cultured for 12–24 hours to the trophozoite stage, as above. Trophozoite-infected RBCs containing paramagnetic hemozoin were enriched to >95% purity by magnetic separation (Miltenyi Biotec, Auburn, CA), inoculated into normal and α-thalassemic RBC samples, and cultured at 1–2% hematocrit as above. In all experiments, parasitized RBCs were assayed after one cycle of invasion and development to the trophozoite stage (∼36 h).

### Endothelial Cell Adherence Assay

Endothelial cell adherence assays were conducted in Mali, except those using HbH RBCs obtained in the United States. Human adult dermal microvascular endothelial cells (MVECs; Lonza, Walkersville, MD) were maintained in the manufacturer’s EGM2-MV medium and grown on LabTek CC-2 coated 8-well chamber slides (Nalge Nunc International, Rochester, NY) to ∼50% confluency at 37°C in a humidified atmosphere of 5% CO_2_. Trophozoite-infected RBCs were adjusted to 5–20% parasitemia and 1% hematocrit by the addition of noninfected RBCs in binding media (BM; RPMI-1640, 0.5% BSA, pH 6.7). Adherent MVECs were washed with BM and then incubated with 150 µl of the parasitized RBC suspension for 1 h at 37°C with constant horizontal agitation (100 rpm). After parasite suspensions were removed from each well, slides were washed by dipping twice in BM at 37°C, fixed in 2% glutaraldehyde at ambient temperature for 2 h, and stained in 1 or 2.5% Giemsa for 30–60 min. In each experiment, the number of parasitized RBCs bound to ∼700 MVECs was counted from duplicate wells. For each slide, the number of parasitized α-thalassemic RBCs per MVEC was normalized to counts from parasitized normal RBCs.

### Monocyte Adherence Assay

Monocyte adherence assays were conducted in Mali, except those using HbH RBCs obtained in the United States. CD14+ monocytes (Lonza) were plated onto CC2 Lab-Tek chamber slides (Nalge Nunc International) at 4×10^5^ cells per well and cultured for 48 h in RPMI 1640 containing 25 mM HEPES, 50 µg/ml gentamicin, and 10% fetal bovine serum at 37°C in an atmosphere of 5% CO_2_. Normal and α-thalassemic RBCs infected with *P. falciparum* trophozoites (3D7, A4tres, FCR-3, naturally-circulating isolates) were purified by magnetic separation, adjusted to 3–15% parasitemia and 1% hematocrit as previously described. Adherent monocytes were washed with binding media and incubated with 150 µl of the parasitized RBC suspension for 1 h at 37°C with gentle horizontal rotation. The parasite suspension was removed from each well and the slides were gently washed by dipping four times in binding media. Slides were dried and stained using Hema 3 (Fischer Scientific). Adherence was measured by counting the number of infected RBCs bound to a minimum of 700 monocytes from duplicate wells. For each slide, the number of parasitized α-thalassemic RBCs per monocyte was normalized to the counts from parasitized normal RBCs.

### Flow Cytometry

Flow cytometry was conducted in the United States using α-thalassemic RBCs obtained in Mali and HbH RBCs obtained in the United States. Rat or rabbit polyclonal antisera raised against PfEMP-1 variants expressed by the *P. falciparum* clones FVO and A4tres were kindly provided by Morris Makobongo and Dror Baruch (NIAID). Rabbit polyclonal antisera specific for a recombinant PfEMP1 variant subdomain (VAR2CSA, DBL3X) was kindly provided by Kavita Singh (NIAID). Samples of trophozoite-infected RBCs (1.5×10^6^ cells; 1% parasitemia) were stained with various dilutions of antiserum in FACS staining buffer (FSB; PBS, 2% FBS, 0.1% sodium azide) for 45 min at ambient temperature and washed twice with FSB. Samples were then incubated with Alexa 488-conjugated anti-rat or anti-rabbit IgG (Molecular Probes, Inc., Eugene, OR) and ethidium bromide (2 µg/ml) at ambient temperature for 30 min and washed twice with FSB. A FACSort instrument (Becton-Dickinson, San Jose, CA) and FlowJo software (Tree Star, Inc., Ashland, OR) were used to acquire and analyze 250,000 to 500,000 events from each sample.

### Atomic Force Microscopy

Parasitized α-thalassemic RBCs were obtained directly from Malian children with malaria, cultured to the trophozoite stage, and prepared for atomic force microscopy (AFM) imaging as described [22]. We also inoculated HbH RBCs with *P. falciparum* clone FCR-3 and cultured them through one cycle of invasion and development to the trophozoite stage (∼36 h). A Bioscope AFM (Veeco Instruments, Santa Barbara, CA) on a wide-field Axiovert 200 fluorescence microscope (Carl Zeiss, Inc., Thornwood, NY) was optimized to image the surface topography of RBCs and to identify the parasite stage within an individual AFM-imaged RBC. The X and Y piezoelectric scanners of the Bioscope AFM were disconnected. A custom built closed-loop XY scanner stage (nPoint, Inc., Madison, WI) was used to minimize scanning artifacts and thermal drift of the scanner for improved image accuracy. AFM was performed in tapping mode in air using Nanosensors pointprobe tips (Nanosensors, Switzerland) with a cantilever resonant frequency of 327–397 kHz. Topographic and error signal (amplitude) images were collected simultaneously. Parasites were stained with YOYO-1 fluorescent nucleic acid staining reagent (Molecular Probes, Inc.). Bright field and fluorescent images were collected with a chilled CCD video camera (Model C5985, Hamamatsu Photonic Systems, Bridgewater, NJ). Image-Pro Plus version 5.0 software (Media Cybernetics, Silver Spring, MD) was used to merge these images to allow the approximate identification of the parasite stage.

### Statistical Analysis

In assays of cytoadherence and PfEMP1 expression, results from parasitized α-thalassemic RBCs were normalized to results from parasitized normal RBCs tested in parallel. 2-tailed *P* values were calculated by one-sample *t* test of the mean using GraphPad software version 5.01 (Graphpad Software, La Jolla, CA).

## Results

**Figure 1:**
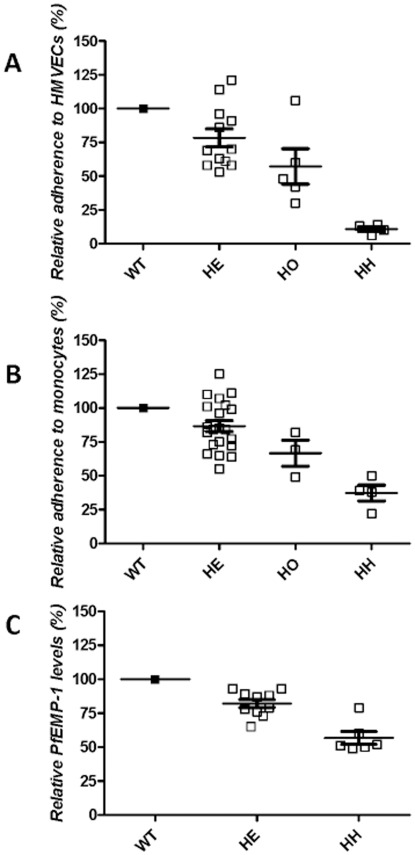
Relative cytoadherence and surface PfEMP1 levels of parasitized RBCs. a, Adherence of parasitized RBCs to MVECs. The numbers of parasitized −α/αα (HE), −α/−α (HO) and −/−α (HH) RBCs adhering to MVECs were normalized to those of parasitized αα/αα RBCs tested in parallel. The mean (± SEM) number of parasitized αα/αα RBCs per 100 MVECs was 260±40, *N* = 19. Results were obtained from 19 naturally-circulating parasite isolates and 2 laboratory-adapted parasite clones (A4tres and FCR-3), multiple blood donors (5 αα/αα, 2−α/αα, 2 −α/−α and 2−/−α), and 4 MVEC donors (not all combinations tested). This resulted in −α/αα, −α/−α and −/−α samples being compared to αα/αα samples 12, 5 and 4 times. **b,** Adherence of parasitized RBCs to monocytes. The numbers of parasitized −α/αα, −α/−α and −/−α RBCs adhering to monocytes were normalized to those of αα/αα RBCs tested in parallel. The mean (± SEM) number of parasitized αα/αα RBCs per 100 monocytes was 136±10, *N* = 12. Results were obtained from 3 naturally-circulating parasite isolates and 3 laboratory-adapted parasite clones (3D7, A4tres and FCR-3), multiple blood donors (5 αα/αα, 3 −α/αα, 2 −α/−α and 2−/−α) and 4 monocyte donors (not all combinations tested). This resulted in −α/αα, −α/−α and −/−α samples being compared to αα/αα samples 20, 3 and 4 times. The αα/αα and −α/αα RBCs were different from those used in endothelial cell adherence assays. **c,** PfEMP1 expression levels (median fluorescence intensities, MFI) on the surface of parasitized RBCs. The mean (± SEM) MFI of parasitized αα/αα RBCs was 556±153, *N* = 6. Results were obtained from 2 laboratory-adapted parasite clones (A4tres, FVO and FCR3^CSA^), multiple blood donors (4 αα/αα, 6 −α/αα and 2−/−α), and various concentrations of 2 antisera (not all combinations tested). This resulted in −α/αα, and −/−α samples being compared to αα/αα samples 10 and 6 times. The αα/αα and −α/αα RBCs were different from those used in endothelial cell and monocyte adherence assays.

Because cytoadherence is centrally involved in the pathogenesis of severe malaria, we tested *P. falciparum*-infected normal and α-thalassemic RBCs for their adherence to MVECs. In these and other cytoadherence comparisons, we used naturally-circulating *P. falciparum* isolates obtained directly from Malian children with malaria, and freshly-drawn nonparasitized RBCs from healthy Malian children. In experiments using −/−α RBCs, we used laboratory-adapted *P. falciparum* clones, and freshly-drawn RBCs from two patients with HbH disease. Relative to parasitized αα/αα RBCs, parasitized −α/αα and −α/−α RBCs showed 22% and 43% reductions in adherence to MVECs, respectively (mean ± SEM; 78%±6.6%, *P* = 0.007, *N* = 12 for −α/αα; 57%±13.0%, *P* = 0.03, *N* = 5 for −α/−α) (**Fig. 1a**). Although the −/−α phenotype is not present in sub-Saharan Africa, we chose to use −/−α RBCs to test the effects of further reductions in α-globin expression on cytoadherence. Parasitized −/−α RBCs showed an 89% reduction in adherence to MVECs (11%±1.8%, *P*<0.0001, *N* = 4) (**Fig. 1a**).

*P. falciparum* infection is associated with elevated levels of TNF and other monocyte-derived cytokines that cause fever and other symptoms of malaria. Excessive levels of these cytokines correlate with the development of severe malaria syndromes [31]; for example, TNF causes dyserythropoiesis that contributes to the development of severe malarial anemia. The interaction between PfEMP1 on parasitized RBCs and CD36 on monocytes leads to monocyte activation and cytokine production *in vitro* [32], [33]. To measure the effects of α-thalassemia on this interaction, we tested the adherence of parasitized RBCs to monocytes. Relative to parasitized αα/αα RBCs, parasitized −α/αα, −α/−α and −/−α RBCs showed 13%, 33% and 63% reductions in adherence to monocytes, respectively (mean ± SEM; 87%±4.0%, *P* = 0.003, *N* = 21 for −α/αα; 67%±9.6%, *P* = 0.07, *N* = 3 for −α/−α; 37%±5.8%, *P* = 0.002, *N* = 4 for −/−α) (**Fig. 1b**).

Reduced expression of PfEMP1 on the surface of parasitized RBCs containing the malaria-protective HbC or HbS variants is associated with reductions in cytoadherence. Using specific antisera in a flow cytometric assay, we found that parasitized –α/αα and −/−α BCs showed 18% and 43% reductions in surface PfEMP1 levels, respectively, relative to parasitized αα/αα RBCs (mean ± SEM; 82%±3.0%, *P* = 0.0002, *N* = 10 for −α/αα; 57%±4.7%, *P* = 0.0003, *N* = 6 for −/−α) (**Fig. 1c**). We were unable to quantify PfEMP1 levels on the surface of parasitized −α/−α RBCs. This is because the homozygous α-thalassemic children in our cohort had confounding polymorphisms such as HbC, HbS and G6PD deficiency, were parasitized at the time of screening, or refused to donate a blood sample.

**Figure 2:**
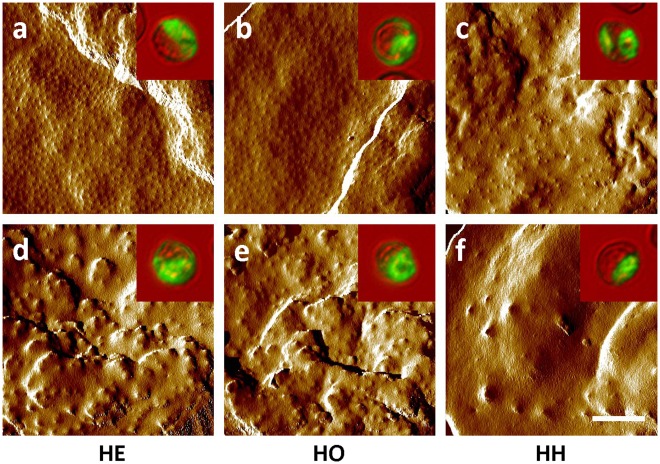
Distribution and morphology of knobs on the surface of parasitized RBCs. Atomic force micrographs (AFMs) of parasitized −α/αα (HE) (**a,d**) and −α/−α (HO) (**b,e**) RBCs obtained from naturally-parasitized Malian children with malaria and −/−α (HH) (**c,f**) RBCs infected with a laboratory-adapted *P. falciparum* clone showing normal (**a,b**) or abnormal (**c–f**) knob distributions and morphologies. AFM images are representative of 32, 10 and 18 images of parasites in −α/αα, −/−αα and −/−α RBCs. Inlays show YOYO-1-stained parasites that correspond to those imaged by AFM. Comparison AFMs of parasitized HbA, HbC and HbS RBCs have been reported previously [22], [37].

On the surface of parasitized RBCs, PfEMP1 proteins are concentrated on knob-like protrusions that make contact with host cells [32], [34], [35]. Alterations in knob assembly can therefore affect the amount and distribution of PfEMP1 [34], [36]. To determine whether reduced surface levels of PfEMP1 are associated with aberrant knob assembly on parasitized α-thalassemic RBCs, we examined the surface of these cells. Atomic force micrographs of naturally-parasitized −α/αα and −α/−α RBCs (cultured *ex vivo* from ring forms to mature trophozoites) showed populations expressing fine, regularly distributed knobs (**Fig. 2a,b**) characteristic of parasitized normal RBCs as well as cell populations expressing large, widely separated knobs (**Fig. 2d,e**) reminiscent of parasitized HbC and HbS RBCs [22], [37]. Knobs on the surface of trophozoite-infected −/−α RBCs were found to be variously abnormal in size and distribution (**Fig. 2c**) and in some cases essentially absent (**Fig. 2f**).

## Discussion

α-thalassemia protects against different clinical forms of severe malaria, especially SMA but also CM and deep acidotic breathing – syndromes associated with fatal outcome. The mechanism by which α-thalassemia protects against these severe malaria syndromes has remained obscure, although it does not seem to affect parasite density *in vivo*. Microcytosis has recently been implicated as a mechanism of protection against SMA by homozygous, but not heterozygous, α-thalassemia in Papua New Guinea [14]. According to this model, increased RBC counts protect homozygotes against SMA by reducing the amount of Hb loss at any given parasite density. While microcytosis might prevent SMA, as defined as Hb concentration ≤50 g/l [14], the role of α-thalassemia homozygosity in protection against death from SMA is less clear. This is because SMA can be associated with very low fatality rates (∼1%) [38] unless it is complicated by other signs of severe malaria, including coma and deep acidotic breathing [4], [38].

To explore how heterozygous and homozygous forms of α-thalassemia protect against CM and complicated SMA, we hypothesized that these traits weaken the adherence of parasitized RBCs to host cells within microvessels – a pro-inflammatory process that is essential in the pathogenesis of severe disease syndromes. We found that progressive reductions in α-globin production produce commensurate reductions in the adherence of parasitized RBCs to MVECs and monocytes. Decreased levels and abnormal distribution of PfEMP1 on the surface of parasitized α-thalassemic RBCs correlate with these findings. Based on these data, we propose the following model to explain how α-thalassemia protects against severe malaria syndromes without affecting parasite densities *in vivo*.

*P. falciparum* merozoites readily invade α-thalassemic RBCs and develop into viable trophozoites expressing PfEMP1. The amount and distribution of PfEMP1 is sufficiently normal to mediate effective sequestration of trophozoite-infected RBCs. This normal cycle of invasion, development, and sequestration enables parasites to avoid clearance from the bloodstream by the spleen and to multiply to densities that are equivalent to those observed in non-thalassemic children with malaria. The avidity of parasitized RBCs for MVECs and monocytes is sufficiently weakened by α-thalassemia, however, so that these host cell populations are not maximally activated. The resulting levels of inflammation produce symptoms of uncomplicated malaria, but are not high enough to cause severe disease and death. This model of protection is consistent with recent data showing that the degree of endothelial cell activation correlates with avidity of cytoadherence [39], [40]. It is also consistent with the finding that parasitized α-thalassemic RBCs show impaired adherence to human umbilical vein endothelial cells and rosetting [41], [42] (the binding of a parasitized RBC to several other noninfected RBCs), a different cytoadherence interaction also implicated in the development of severe disease.

Our model suggests that α-thalassemia heterozygous and homozygous states confer protection against CM and complicated SMA by impairing PfEMP1-mediated activation of host cells. By impairing endothelial cell activation, for example, α-thalassemia could dampen the degree of endothelial dysfunction that contributes to CM. By impairing monocyte activation, α-thalassemia could produce lower levels of cytokines and other inflammatory mediators that suppress erythropoiesis [43]. Some evidence for this possibility was recently provided by Veenemans *et al*. who showed that in Tanzanian and Kenyan children, α-thalassemia limits the decline in Hb levels associated with episodes of symptomless parasitemia – particularly those accompanied by inflammation [8]. The lower mean corpuscular Hb concentrations associated with α-thalassemia may contribute to this protective mechanism by decreasing the amount of free Hb released upon the rupture of each schizont-infected RBC. This mechanism would have the effect of lessening the severity of free Hb-induced endothelial cell dysfunction, as proposed by May *et al*. [4].

Our model also provides a plausible explanation for how heterozygous α-thalassemia confers less protection against severe disease than do HbS and HbC. Indeed, large numbers of α-thalassemic children *do develop severe disease* despite their abnormal RBC phenotype. Multiple studies have reported significant numbers of α-thalassemic children who develop life-threatening manifestations of disease [(e.g., 31% (94/301) of Ghanaian children in [2]; 60% (391/655) of Kenyan children in [3]; and 51% (70/137) of Kenyan children in [13])]. Relatively mild reductions in surface levels of particular PfEMP1 variants may not suffice to significantly impair the avidity of parasitized RBCs for host cells, resulting in relatively significant proportions of children with severe disease carrying the heterozygous α-thalassemia phenotype. A major limitation of our study is that it used −α/−α RBCs from relatively few donors. While data from −α/−α (and HbH) RBC samples were useful to explore the effect of α-thalassemia dose on PfEMP1 display, additional laboratory and epidemiological studies are needed to determine the relative contribution of impaired cytoadherence to the malaria-protective effects of homozygous α-thalassemia.

Parasitized α-thalassemic RBCs show PfEMP1 display abnormalities that are reminiscent of those on the surface of parasitized HbS and HbC RBCs. Shared characteristics between HbS, HbC, and α-thalassemic RBCs suggest possible mechanisms by which these diverse hemoglobinopathies impair the ability of *P. falciparum* parasites to remodel their host RBC membrane. HbS, HbC, and unpaired β-globin chains (in the case of α-thalassemia) undergo accelerated degradation to hemichromes [44], [45]. These hemichromes bind the inner leaflet of the RBC membrane where they promote the heme iron-mediated oxidation of membrane proteins and lipids [46], [47] – a process that consumes antioxidants as well. Since parasites induce hemichrome formation as they mature from ring to trophozoite stages [48], the amount of hemichromes produced by parasites in HbS, HbC, and α-thalassemic RBCs would be greater than in normal RBCs [49]. Excessive hemichrome-induced damage to the parasitized RBC membrane could interfere with the trafficking and knob incorporation of PfEMP1.

Finally, our data suggest that α-thalassemia protects against severe *P. falciparum* malaria by the same mechanism as HbS and HbC: ameliorating the pro-inflammatory effects of cytoadherence. This model also raises the possibility that other unstable hemoglobins such as HbE and unpaired α globin chains (in the case of β-thalassemia) protect against severe malaria by a similar mechanism. While appropriate case-control and cohort studies have not yet been conducted to determine whether HbE or β-thalassemia confer protection against fatal malaria syndromes, neither hemoglobinopathy has been associated with reduced parasite prevalence or densities *in vivo* [50], [51], [52]. We speculate that diverse hemoglobinopathies have been naturally selected worldwide for the common phenotype of abnormal PfEMP1 display. If true, these findings would suggest that PfEMP1-mediated phenomena are centrally responsible for malaria-related deaths and that therapeutics and vaccines that interfere with cytoadherence may reduce malaria mortality.
